# Magnitude of Anemia and Hematological Predictors among Children under 12 Years in Odisha, India

**DOI:** 10.1155/2016/1729147

**Published:** 2016-04-04

**Authors:** Shuchismita Behera, Gandham Bulliyya

**Affiliations:** Regional Medical Research Centre, Indian Council of Medical Research, Bhubaneswar 751023, India

## Abstract

*Background*. Anemia is a wide spread public health problem in India which affects children. The present study evaluates the prevalence of anemia and status of various hematological parameters among children of Khurda district, Odisha.* Method*. A total of 313 children aged 0–12 years were enrolled for the study which included preschool (0–5 years) and school aged (6–12 years) groups. Hematological indicators were measured by standard procedures, which include red blood cell (RBC) indicators, white blood cell (WBC) indicators, and plasma ferritin.* Results*. Mean hemoglobin (Hb) of the study population was 10.43 ± 3.33 g/dL and prevalence of anemia was 62%. In this population, boys had a lower mean Hb value than that of the girls. All grades of anemia were higher among school age children than preschool children. Mean plasma ferritin was found to be higher in school age boys than their counterpart girls. The mean level of WBC count was found to be higher among preschool age boys than among the school age boys (*p* = 0.025).* Conclusion*. The prevalence of anemia was higher with concomitant acute infection among study population, which is a matter of concern. Since the hematological parameters are interrelated with each other as well as with the age and gender, relevant intervention strategy and constant monitoring are needed while providing public health nutrition programs to eradicate anemia.

## 1. Introduction

Anemia is a widespread public health problem associated with an increased risk of morbidity and mortality, especially in pregnant women and young children [1]. Globally 1.62 billion people are anemic, while among the preschool children the prevalence of anemia is 47.4%. Nutritional anemia in South Asia accounts for nearly half of global cases of anemia. In India, anemia continues to be the major health problem in young children, adolescent girls, and pregnant women. Approximately 50% of the population suffers from nutritional anemia as known in countries where meat consumption is low [2].

In India, about 89 million children are anemic. The prevalence of anemia was 70% in children aged 6–59 months [3]. The highest prevalence of anemia was seen in children <10 years, especially in those <5 years [4]. Iron deficiency is one of the most common causes of anemia [5]. Besides iron, other nutrients such as vitamins A, E, and C also play key role in formation and protection of red blood cell (RBC) by stimulating stem cells as well as by activating a number of antioxidant enzymes [6]. Therefore inadequacy of any of these micronutrients may lead to anemia in the vulnerable sections of population. Studies have shown that preschool children are more vulnerable to the risk of iron deficiency anemia. The prevalence of iron deficiency anemia is the highest among preschool children. In this age group (6–59 months), body grows rapidly and requires high-iron-rich and nutritious food that may not be fulfilled by their normal diet. Low economic status, less education, and poor health of mothers due to meager dietary intake are the main causes of anemia. Anemia is the most predominant factor for morbidity and child mortality, and, hence, it is a critical health issue for children in India. Iron deficiency affects cognitive and motor development and increases susceptibility to infections. The prevention as well as timely management of anemia is essential to attain Sustainable Development Goal-3 (SDG) on ensuring healthy lives and promoting wellbeing for all at all ages. Further actions are required to reach the World Health Assembly target of a 50% reduction of anemia in women of reproductive age by 2025.

Odisha (formerly Orissa) is one of eight empowered action group (EAG) states of India with poor demographic and socioeconomic indicators including maternal and child health. The Clinical, Anthropometric, and Biochemical (CAB) survey conducted recently in 2014 shows that 70.6% and 81.2% of children aged 6–59 months and 5–9-year-old children are suffering from anemia. Hence, information regarding young children is inadequate on factors affecting anemia. However, no report is available on prevalence of anemia among children in Khurda district. In the present study, an attempt has been made to assess the magnitude of iron deficiency anemia by measuring hematological indices.

## 2. Materials and Methods

### 2.1. Setting

The study was conducted in the rural surroundings of Bhubaneswar city in Khurda district, the state capital of Odisha located on the east coast of India, by the Bay of Bengal. Apparently healthy children aged less than 12 years were chosen for the study, which included preschool children (0–5 years) and school age children (6–12 years). Children having medication in the past fortnight prior to data collection and unwilling individuals were excluded from the study.

### 2.2. Study Design

This is a cross-sectional community-based survey. All children and their parents were informed about the purpose and the method of the research and the voluntary nature of participation in the study verbally and in written form.

### 2.3. Ethical Consideration

 Informed written consent was obtained from the parents of each child after the study objective was explained. The study protocol was approved by the Institutional Human Ethical Committee of Regional Medical Research Centre, Bhubaneswar.

### 2.4. Data Collection

A pretested questionnaire was applied to obtain relevant information of demographic and socioeconomic data. Age of each child was collected from date of birth certificate or birth records available with mother. Confirmation of a child's age was made with the mother with the help of Anganwadi Workers, community health workers.

### 2.5. Anthropometric Measurements

Body weight and height were measured using standardised equipment and procedures. Body mass index (BMI) for each child was calculated based on the ratio of weight (kg) to height in square meters. BMI data were transformed to *z*-scores, namely, BMI-for-age *z*-score (BAZ) using the WHO Growth Standards [7].

### 2.6. Blood Samples

Either finger prick or venous blood was collected according to the agreement of the participants. The finger prick blood was transferred to Whatman number 1 filter paper while two mL of venous blood was dispensed into vials containing EDTA anticoagulant bottles. Hematological and biochemical investigations were carried out in nutrition laboratory.

### 2.7. Biochemical Estimations

Venous blood was subjected to complete blood count (CBC), which was performed by an automated analyzer MS4 (Melet Schloesing 4, Germany) used for the* in vitro* diagnostic testing. The blood was well mixed (though not shaken) and placed on a rack in the analyzer. The instrument counted the number and type of different cells within the blood and results were printed out that included hemoglobin (Hb), red blood cell (RBC), hematocrit (HCT), mean corpuscular volume (MCV), mean corpuscular hemoglobin (MCH), mean corpuscular hemoglobin concentration (MCHC), red cell distribution width (RDW), white blood cell (WBC), granulocyte, thrombocyte (platelet), lymphocyte, and monocytes. Adequate quality control measures were taken on each test procedure to ensure the reliability of the results. The validity of Hb measure was confirmed by checking the reproducibility of the results of sample aliquots by cyanmethemoglobin method. The finger prick blood was used to estimate hemoglobin (Hb) by cyanmethemoglobin method [8]. Plasma ferritin was estimated using ELISA kits obtained from United Biotech Inc. Magiwell Ferritin, USA (K951993).

### 2.8. Statistical Analysis

Anemia was defined as Hb concentration <11 g/dL for children aged between 6 and 59 months while <11.5 g/dL for children aged between 5 and 11 years and <12 g/dL for children aged 12 years according to WHO as shown in Table 1 [9]. Further, normal reference ranges used for hematological indicators (red blood indices and white blood indices) are provided in Table 2 [10, 11]. Data were entered in Microsoft Excel 2007 and all statistical analyses were performed with GraphPad Prism software (version 4.00). SPSS for Windows version 17.0, Chicago, USA, was also used for data analysis. Descriptive characteristics (mean and standard deviation) and percentage were performed for each parameter separately. Chi-square and independent *t*-test were used for proportions and mean comparisons between groups, respectively. Pearson's correlation tests were performed to examine the relationships between hematological indicators. The strength of association is measured by unadjusted odds ratio (OR) and 95% confidence interval (CI).

## 3. Results

A total of 313 children provided blood samples for estimation of Hb, ferritin (250), hematological indicators (139), and WBC differential count (131). The mean characteristics of BAZ and hematological parameters are shown in Table 3. The mean BAZ of study population was −1.04 ± 1.53 and no significant difference was observed between the age or gender groups. The mean Hb of the study population was 10.43 ± 3.33 g/dL. The mean Hb among preschool children was 10.45 ± 2.99 g/dL of which boys had 10.57 ± 3.01 g/dL and girls had 10.27 ± 2.99 g/dL. The mean Hb among school age children was found to be 10.42 ± 3.491 g/dL of which boys had 9.78 ± 3.96 g/dL and girls had 10.85 ± 3.98 g/dL. Hb, HCT, MCV, and MCH of school age boys were significantly lower than girls (*p* = 0.029, 0.042, 0.0002, and 0.023, resp.). The mean ferritin level among boys was significantly higher than girls (*p* = 0.0002), which was chiefly exhibited by school age children. The mean WBC count was found to be higher among preschool boys than among school age boys (*p* = 0.025). Mean MCV and MCH were significantly higher among school age girls than preschool girls (*p* = 0.026 and 0.011, resp.). Also, MCH and MCHC of preschool children were low compared to school children (*p* = 0.009 and 0.006, resp.). Mean RDW of preschool girls was higher than their male counterpart as well as school age girls (*p* = 0.016 and 0.026).

The prevalence of different grades of anemia according to Hb level is depicted in Table 4. Overall occurrence of anemia was 62% comprised of 23% mild, 23% moderate, and 16% severe categories. School age children were found to be more anemic than preschool children. Prevalence of anemia was 48.5% among preschool children of which 47.6% were boys and 50.0% were girls. Mild, moderate, and severe anemia were found to be 12.9%, 22.7%, and 12.9%, respectively. Among school age children 68.9% had anemia, of which 27.4% were mildly, 23.6% were moderately, and 17.9% were severely anemic. Girls were significantly more anemic than boys in the age group of 11-12 years (93.1% versus 72.2%, *p* = 0.028) while more boys were anemic in the age group 10-11 years (84.6% versus 53.1%, *p* = 0.048).

Table 5 shows the hematological indicators for anemia with standard reference ranges. Deficiency of RBC count was found to be 47.5% in children indicating iron, vitamin B12, or folate deficiency or hemolysis. Further, deficiency of HCT was 52.5% indicating the same. Deficiency of MCV, MCH, and MCHC was 15.1%, 30.9%, and 55.4%, respectively, demonstrating a probable iron deficiency while value above the reference level for MCV and MCH indicates probability of vitamin B_12_ deficiency for 14.4% and 5.7%, respectively. Microcytic anemia in terms of MCV and MCH was higher among school age boys than girls. Although deficiency of RDW (<11%) was 59.7%, it does not signify to a concerning problem; however, a higher cut-off value indicates an iron/vitamin B_12_/folate deficiency of 11.5% (RDW > 15%).

Table 6 represents WBC indicators and thrombocyte according to standard reference ranges. The prevalence of WBC deficiency was 35.3% indicating immunosuppression/viral infection. About 12.2% of children had WBC level above the cut-off level to represent inflammation or infection. The prevalence of granulocyte, monocyte, and lymphocyte deficiency was 64.9%, 32.8%, and 0.8%, respectively, which is indicative of immunosuppression. High level of granulocyte was prevalent in 1.5% of children indicating infection/inflammation. About 6.9% of children had high monocyte count demonstrating chronic infection while 74.8% had high lymphocyte count reflecting their susceptibility to viral infections. About 63.4% of children were subclinically deficient for thrombocyte (platelet), which is an important blood-clotting factor. Higher level of thrombocytes observed in 7.6% of children is an indicative sign of viral infection/pernicious anemia.

The relationship between different health indicators of children is analyzed by correlation to establish the degree of association (Table 7). Hb, HCT, and RBC count were positively correlated with BAZ while the latter was negatively correlated with RDW. Hb was positively correlated with RBC, HCT, MCV, MCH, and monocyte but inversely correlated with WBC, RDW, and granulocyte. WBC was negatively correlated with HCT and lymphocyte and positively correlated with thrombocyte and granulocyte. RBC was positively correlated with HCT and monocyte and inversely correlated with MCH and RDW. Similarly HCT was well correlated with MCV and monocyte and negatively correlated with RDW. MCV was found to be associated with MCH and monocyte and negatively correlated with RDW and granulocyte. RDW is negatively correlated with MCH and monocyte. Granulocyte was positively correlated with MCHC and negatively correlated with monocyte and lymphocyte. Monocyte was negatively correlated with MCHC.

## 4. Discussion

The present study attempted to assess the association of hematological indices with the prevalence of anemia among children in the rural surroundings of Bhubaneswar city, India. The mean Hb among school age boys was significantly lower than girls. Sahu et al. [12] also found a lower mean Hb level in school age boys than girls in Gajapati district, Odisha. Bulliyya et al. [13] showed a mean Hb level of 10.07 among adolescent girls of Khurda district, Odisha.

The prevalence of anemia among preschool children was 48.5%, which is much less when compared to the state data of 92.4% [14]. The type of anemia among school age children was 68.9% (mild 27.4%, moderate 23.6%, and severe 17.9%). Sahu et al. [12] found severity of anemia (35.2% mild, 59.4% moderate, and 5.4% severe) in children in Gajapati district much higher than the value in this study. Girls were significantly more anemic than boys in the age group of 11-12 years while more boys were anemic in the age group of 10-11 years. Similar results were reported for school children in Bangalore where prevalence of anemia was higher in boys aged 10 years whereas it was high in girls aged 11 years [15].

The mean HCT, MCV, and MCH of school age boys were significantly lower than girls'. Zemel et al. [16] observed a significantly lower HCT among boys than girls of school age sickle cell children (excluding children receiving transfusion therapy). In this study chronic undernutrition (stunting) may be one of the factors for lower level of HCT. Kokore et al. [17] found that MCV and MCH are statistically higher for girls than their male counterparts aged 5–11 years. The hypochromasia (MCH deficient) and microcytosis (MCV deficient) in school age population are higher in boys than in girls. The disruption of erythrocyte parameters like MCV and MCH precedes the final stage of anemia with concurrent fall in Hb levels below the limit. In this study, decrease in MCV and MCH might indicate a deficiency in micronutrients including iron and vitamins as suggested earlier [18].

Mean MCV and MCH were significantly higher among school age girls than preschool girls. Moreover, MCH and MCHC of preschool children were low compared to school children. Similar findings were observed among girls of different age groups [19]. It was found that MCV and MCH were slightly lower in those under-5 children but subsequently increased and reached to the adult level by age of 6 years [20]. Several studies reported an increase in mean MCH and MCHC levels with increase in age [19, 21]. Vitamin B_12_ deficiency in terms of value above reference level for MCV and MCH was found to be 14.4% and 5.7%, respectively. Bleyere et al. [19] reported 5.1% of probable vitamin B_12_ deficiency (high MCV) among children.

In the current study, mean WBC count and proportion above the upper level were higher among preschool boys than school age boys (13.8% versus 5.9%). Excess WBC in the peripheral blood may be indicative of various disease states, including inflammation (acute or chronic) from bacteria virus or parasites [22]. Porniammongkol et al. [23] demonstrated that the percentage of children with elevated WBC compared to normal range was higher in the younger age than in the older age. In this study, the prevalence of WBC deficiency was 35.3% indicating immunosuppression/viral infection while 12.2% of them had WBC level above the cut-off level representing inflammation or infection. Bleyere et al. [19] found WBC level below the range in 26.8% and 0.8% in above the range of children in West Africa. The percentage of children above the upper limit was much higher in this study population and the reason may be that both WBC and granulocyte are inversely correlated with Hb (*p* < 0.001). It was also previously well documented that WBC and percentage of neutrophil are inversely associated with Hb [24]. Since a large proportion of study population is anemic (62%) that may lead to overall elevated levels of WBC.

The mean RDW was 11.5% in the study population of which preschool girls have significantly higher level of RDW both by mean level and frequency. It was previously demonstrated that RDW levels were significantly higher in lower age group of iron deficient children in Turkey as well as girls who had higher value than boys [25]. A higher cut-off value for RDW indicates an iron/vitamin B_12_/folate deficiency observed to be 11.5% in the study population. An elevated RDW is also believed to be an early indicator of iron deficiency [26].

The mean values of granulocyte, monocyte, and lymphocyte were 36.71%, 5.68%, and 57.38%, respectively, and preschoolers had correspondingly 36.16%, 6.02%, and 57.14% as confirmed by our previous study [27] along with school age children having 37.00%, 5.50%, and 57.50%, respectively. The mean monocyte and lymphocyte values in children of West Africa were found to be 5.2% and 51.3%, respectively [17], which is closer to our value. The prevalence of granulocyte, monocyte, and lymphocyte deficiency was 64.9%, 32.8%, and 0.8%, respectively. About 74.8% of children had high lymphocyte count representing their susceptibility to viral infection. Kokore et al. [17] found only 0.3% of lymphocyte deficiency in children, which is much less than that of our value, while a high lymphocyte count was observed among 88.7% of children. In this study chronic infection was indicated by high monocyte count among 6.9% of children. The prevalence of low monocytes is reported to be less at 6.5% and 4.8%, respectively, in other studies [17, 19]. Monocytes represent a source of proinflammatory cytokines and thus are believed to play a role in obesity-associated disease [28]. Chapman et al. [29] demonstrated monocyte concentration to be an independent risk factor for subclinical carotid atherosclerosis. About 63.4% of children were below the lower range for thrombocyte. Higher level of thrombocytes observed in 7.6% of children indicates viral infection/pernicious anemia. This high number of platelet deficiency may be due to various cut-off levels suggested to be used for different age group of children whereas many authors recommended to consider a single cut-off for all age groups (<150 thousand/mm^3^). Using this range, Kokore et al. [17] and Bleyere et al. [19] found low thrombocytes in 1.9% and 5.8% of total population, respectively. It was observed that disorders of the bone marrow and other medical conditions could cause an elevated platelet count [30].

Hb was positively correlated with RBC, HCT, MCV, MCH, and monocyte in this population. At birth, the total Hb level, RBC, and HCT are shown to be higher than at any other period of life [31]. The Hb content and the RBCs then gradually rise to adult levels by the age of puberty [32]. Maude et al. [33] even also found RBC correlated positively with total Hb in homozygous sickle cell patients where there is abnormal synthesis of Hb. It was established that the HCT usually correlates well with Hb but is even less sensitive for iron deficiency than Hb [34]. The positive association between Hb and MCV suggests a lesser chance of macrocytic anemia in the study population as the concentration of Hb varies concomitantly with cell volume. When RBCs divide in the bone marrow compartment, the resultant two daughter cells after each division are slightly smaller than the parent cell. The reduction in the number of such divisions results in the eventual erythrocytes being larger than usual or macrocytic, with a raised MCV leading to an overall reduction in cell division and in a reduction in Hb biosynthesis [35]. Khan et al. [36] found significant relationship between Hb and MCH in elderly Pakistani males. Under iron deficiency condition, formation of Hb is reduced resulting in a reduction of MCH [37]. The transmembrane protein (ferroportin) is responsible for the transfer of iron from enterocytes and monocytes/macrophages to the circulation [38]. It was found that ferroportin mRNA expression was significantly reduced in monocytes of anemic subjects compared with controls [39]. Importantly, the decreased expression of ferroportin was paralleled by increased iron storage in monocytes of anemia of chronic disease patients as estimated by hyperferritinemia. As a functional consequence of decreased ferroportin expression and the subsequent reduction of cellular iron export, intracellular iron levels will increase which interferes in the process of erythropoiesis, thus decreasing expression of monocytes leading to decrease in Hb.

In this study, RDW was negatively correlated with Hb, RBC count, HCT, MCV, MCH, and monocyte. Lippi et al. [40] also found RDW negatively correlated with Hb and MCV, while inverse relationship of RDW was seen with the Hb in iron deficiency anemia; however, no such correlation happened with noniron deficiency anemia [41]. RDW is shown to have weak inverse correlation with that of HCT, Hb, and MCV in animal model [42] and humans [43]. The RDW is usually increased in macrocytosis. Occasionally in spherocytosis and polychromatic macrocytes there are small red cell agglutinates or red cells that have been ingested by monocytes [44]. Increased RDW indicates the presence of anisocytosis, which is related to impaired erythropoiesis and erythrocyte degradation, reflecting chronic inflammation and a high level of oxidative stress [45].

RBC was associated with HCT and monocyte and inversely with MCH. In anemia, a reduction of the Hb is usually accompanied by reduction in the RBC and HCT [44]. It was revealed that venous HCT values correlated highly with circulating RBC volume [46]. Under autologous experimental conditions, the presence of oxidative stressed erythrocytes in blood exacerbates cytokine production markedly and thus the activation status of human monocytes indicates a probable influence of oxidative stress in these children [47]. In anemic condition a marked fall in RBC, Hb, and HCT and a parallel increase in the MCV and MCH were observed [44].

HCT was positively correlated with MCV and monocyte. Weir and Scott [35] noticed a positive correlation between HCT and MCV among elderly Pakistani males. MCV was found to be associated with MCH and monocyte positively and negatively with the granulocyte. There is a strong association between MCV and MCH in iron deficiency and megaloblastic conditions [48]. It was observed that association between high MCV (above cut-off) and high MCH (above cut-off) as well as a higher peripheral blood monocyte count with venous thrombosis indicates association of these blood indices* in vivo* [49].

WBC was negatively correlated with HCT and lymphocyte and positively with thrombocyte and granulocyte. It is established that HCT among children of 2–16 years is significantly lower among cases of bacterial and viral infection where WBC is higher than controls [24]. WBC was correlated with thrombocyte in the adolescent population [50]. Jabeen et al. [51] also noticed negative correlation of WBC with lymphocyte but positive correlation with granulocyte. Granulocyte was correlated negatively with monocyte and lymphocyte in tandem with others [51]. MCHC was inversely associated with monocyte and positively with granulocyte. The correlation between HCT and monocyte (*p* < 0.001) is more significant than the correlation between Hb and monocyte (*p* < 0.01). As MCHC is the ratio between Hb and HCT, negative correlation with monocyte indicates that with increase in monocyte percentage there is increase in Hb, but the rate of increment is less than that of HCT, which is also evidenced by their strength of association.

School age boys had significantly higher mean levels of ferritin than school age girls. It was observed that, beginning in adolescence, males have higher values of ferritin than females, a trend that persists into late adulthood [52]. The positive correlation of BAZ with Hb, RBC count, and HCT shows that rural children suffer more chronic malnourishment coupled with anemia [53]. Higher RDW is associated with systemic inflammation and undernutrition and represents an integrative measure of the pathological process and hence a negative relation between BAZ and RDW [54].

## 5. Conclusion

The present study showed magnitude of anemia among children, which emphasizes the fact that existence of 62% anemia in the population is a matter of concern which is also related to undernutrition. The prevalence of high lymphocyte count in the population also indicates viral infection. The prevalence of anemia was higher in lower age group, which was further more due to frequent infections. Girls of preschool age showed a probable iron, vitamin B_12_, or folate deficiency as indicated by high RDW value. Girls of the adolescent age (11-12 years) were more anemic indicating more nutritional requirement with the onset of puberty. However, overall school age boys were found to be suffering from higher level of hypochromasia and microcytic anemia. Since the hematological parameters are interrelated with each other as well as with the gender and age groups, constant monitoring and intervention strategy is needed while providing nutritional supplementation to eradicate anemia. We recommend awareness creation on water and sanitation and nutritional counselling to parents on consumption of iron-rich foods and iron supplementation to prevent anemia among young children with special emphasis on those from low income group and socioeconomic deprived communities.

## Figures and Tables

**Table 1:** Hemoglobin concentrations (g/dL) for the diagnosis of anemia and assessment of severity according to the WHO/UNICEF/UNU (2001).

Anemia measured by hemoglobin (g/dL)				
	Anemia	Mild	Moderate	Severe
Children 6–59 months	<11.0	10–10.9	7.0–9.9	<7.0
Children 5–11 years	<11.5	10–11.4	7.0–9.9	<7.0
Children 12–14 years	<12.0	10–11.9	7.0–9.9	<7.0

**Table 2:** Reference range/cut-off values assigned for different hematological parameters.

Hematological parameters	Age group	Reference value	Reference
Red blood count (million/mm^3^)	6 months–2 years	3.7–5.3	[10]
	2 years–6 years	3.9–5.3	
	6 years–12 years	4.0–5.2	
Hematocrit (%)	6 months–2 years	33–39	
	2 years–6 years	34–40	
	6 years–12 years	35–45	
Mean corpuscular volume (fl)	6 months–2 years	70–86	
	2 years–6 years	75–87	
	6 years–12 years	77–95	
Mean corpuscular hemoglobin (pg)	6 months–2 years	23–31	
	2 years–6 years	24–30	
	6 years–12 years	25–33	
Mean corpuscular hemoglobin concentration (g/dL)	6 months–2 years	30.0–36.0	
	2 years +	32.3–35.7	
White blood counts (thousand/mm^3^)	6 months–2 years	6.0–17.0	
	2 years–4 years	6.0–15.5	
	4 years–6 years	5.5–14.5	
	6 years–12 years	4.5–13.5	
Thrombocytes (thousand/mm^3^)	0-1 month	250–450	
	1 month–1 year	300–750	
	1–3 years	250–600	
	3–7 years	250–550	
	7–12 years	200–450	

Granulocytes (%)	All age groups	18–45	[11]
Monocytes (%)	All age groups	4–11	
Lymphocytes (%)	All age groups	45–75	
Red cell distribution width (%)	All age groups	11–15	

**Table 3:** Mean BMI-for-age *z*-score and hematological parameters among children in Odisha, India.

Variables	Sex	*N*	Preschool children	*N*	School age children	*N*	Pooled children
BMI-for-age *z*-score	Boys	63	−0.96 ± 1.82	85	−0.96 ± 1.59	148	−0.96 ± 1.68
	Girls	38	−0.87 ± 1.34	127	−1.18 ± 1.37	165	−1.11 ± 1.368
	Total	101	−0.93 ± 1.65	212	−1.09 ± 1.46	313	−1.04 ± 1.53

Hemoglobin (g/dL)	Boys	63	10.57 ± 3.01	85	9.78 ± 3.96	148	10.11 ± 3.59
	Girls	38	10.27 ± 2.99	127	10.85 ± 3.09^*∗*^	165	10.71 ± 3.06
	Total	101	10.45 ± 2.99	212	10.42 ± 3.49	313	10.43 ± 3.33

Plasma ferritin (ng/mL)	Boys	49	177.9 ± 226.8	75	279.5 ± 280.0^$^	124	239.4 ± 264.0
	Girls	23	162.1 ± 218.2	103	122.8 ± 176.3^*∗*^	126	130.0 ± 184.4^*∗*^
	Total	72	172.9 ± 222.6	178	189.2 ± 238.4	250	184.5 ± 233.6

White blood cell count (thousand/mm^3^)	Boys	29	9.51 ± 6.43	51	6.72 ± 4.44^$^	80	7.74 ± 5.38
	Girls	17	8.12 ± 5.45	42	8.03 ± 7.13	59	8.05 ± 6.64
	Total	46	8.99 ± 6.06	93	7.31 ± 5.81	139	7.87 ± 5.93

Red blood cell count (million/mm^3^)	Boys	29	4.21 ± 1.16	51	3.69 ± 1.29	80	3.88 ± 1.26
	Girls	17	3.823 ± 1.71	42	4.02 ± 1.59^*∗*^	59	3.96 ± 1.61
	Total	46	4.07 ± 1.38	93	3.84 ± 1.43	139	3.92 ± 1.41

Hematocrit (%)	Boys	29	35.20 ± 10.25	51	29.94 ± 10.93^$^	80	31.85 ± 10.93
	Girls	17	29.52 ± 12.60	42	35.33 ± 14.26^*∗*^	59	33.65 ± 13.95
	Total	46	33.10 ± 11.38	93	32.37 ± 12.76	139	32.61 ± 12.28

Mean corpuscular volume (fl)	Boys	29	83.58 ± 6.96	51	81.35 ± 8.11	80	82.16 ± 7.74
	Girls	17	81.15 ± 14.09	42	88.01 ± 8.61^*∗*$^	59	86.04 ± 10.82^*∗*^
	Total	46	82.68 ± 10.11	93	84.36 ± 8.94	139	83.81 ± 9.34

Mean corpuscular hemoglobin (pg)	Boys	29	25.41 ± 2.72	51	26.18 ± 3.63	80	25.90 ± 3.33
	Girls	17	24.76 ± 5.32	42	27.95 ± 3.73^*∗*$^	59	27.03 ± 4.44
	Total	46	25.17 ± 3.84	93	26.98 ± 3.76^$^	139	26.38 ± 3.87

Mean corpuscular hemoglobin concentration (g/dL)	Boys	29	30.48 ± 2.35	51	32.29 ± 3.39^$^	80	31.63 ± 3.16
	Girls	17	30.89 ± 2.83	42	31.82 ± 2.52	59	31.55 ± 2.62
	Total	46	30.63 ± 2.52	93	32.08 ± 3.02^$^	139	31.60 ± 2.93

Red cell distribution width (%)	Boys	29	10.79 ± 1.56	51	11.73 ± 3.00	80	11.39 ± 2.60
	Girls	17	13.28 ± 5.04^*∗*^	42	11.01 ± 2.58^$^	59	11.66 ± 3.58
	Total	46	11.71 ± 3.47	93	11.40 ± 2.83	139	11.50 ± 3.05

Granulocyte (%)	Boys	29	36.40 ± 17.49	48	38.12 ± 17.28	77	37.49 ± 17.26
	Girls	17	35.76 ± 18.35	37	35.54 ± 17.52	54	35.61 ± 17.61
	Total	46	36.16 ± 17.61	85	37.00 ± 17.33	131	36.71 ± 17.37

Monocyte (%)	Boys	29	6.76 ± 4.75	48	5.44 ± 4.02	77	5.92 ± 4.32
	Girls	17	4.81 ± 2.30	37	5.59 ± 3.47	54	5.34 ± 3.15
	Total	46	6.02 ± 4.08	85	5.50 ± 3.77	131	5.68 ± 3.87

Lymphocyte (%)	Boys	29	55.88 ± 15.28	48	56.45 ± 17.58	77	56.24 ± 16.67
	Girls	17	59.22 ± 17.62	37	58.87 ± 17.34	54	58.98 ± 17.26
	Total	46	57.14 ± 16.09	85	57.50 ± 17.41	131	57.38 ± 16.91

Thrombocyte count (thousand/mm^3^)	Boys	29	331.2 ± 450.6	48	229.9 ± 233.3	77	267.7 ± 332.2
	Girls	17	222.2 ± 164.1	37	205.4 ± 149.2	54	210.7 ± 152.7
	Total	46	290.0 ± 370.5	85	219.1 ± 199.9	131	243.8 ± 272.5

^*∗*^ *p* < 0.05 for boys versus girls; ^$^ *p* < 0.05 for preschool versus school children.

**Table 4:** Prevalence (%) of anemia by hemoglobin (g/dL) among children in Odisha.

Age group (years)	Sex	*N*	Anemia grade				Total anemic	*p* value	OR (95% CI)
			Normal	Mild	Moderate	Severe			
Preschool children	Boys	63	52.4 (33)	12.7 (8)	22.2 (14)	12.7 (8)	47.6 (30)	0.816	1.10 (0.49–2.46)
	Girls	38	50.0 (19)	13.2 (5)	23.6 (9)	13.2 (5)	50.0 (19)		
	Total	101	51.5 (52)	12.9 (13)	22.7 (23)	12.9 (13)	48.5 (49)		

School age children	Boys	85	27.1 (23)	17.6 (15)	25.9 (22)	29.4 (25)	72.9 (62)	0.295	1.38 (0.75–2.52)
	Girls	127	33.9 (43)	33.9 (43)	22.0 (28)	10.2 (13)	66.1 (84)		
	Total	212	31.1 (66)	27.4 (58)	23.6 (50)	17.9 (38)	68.9 (146)		

Pooled children	Boys	148	37.8 (56)	15.6 (23)	24.3 (36)	22.3 (33)	62.2 (92)	0.962	1.01 (0.64–1.59)
	Girls	165	37.6 (62)	29.1 (48)	22.4 (37)	10.9 (18)	62.4 (103)		
	Total	313	37.7 (118)	22.7 (71)	23.3 (73)	16.3 (51)	62.3 (195)		

OR = odds ratio, CI = confidence interval; figures in parentheses are sample number.

**Table 5:** Distribution of hematological (red blood cell) indicators by age group and sex among children in Odisha, India.

Parameter	Category	Preschool children			School age children			Pooled children		
		Boys	Girls	Total	Boys	Girls	Total	Boys	Girls	Total
Red blood cell count (million/mm^3^)	Normal	75.9 (22)	47.1 (8)	65.2 (30)	47.1 (24)	45.2 (19)	46.2 (43)	57.5 (46)	45.8 (27)	52.5 (73)
	Deficiency	24.1 (7)	52.9 (9)	34.8 (16)	52.9 (27)	54.8 (23)	53.8 (50)	42.5 (34)	54.2 (32)	47.5 (66)

Hematocrit (%)	Normal	65.5 (19)	47.1 (8)	58.7 (27)	35.3 (18)	50.0 (21)	41.9 (39)	46.2 (37)	49.2 (29)	47.5 (66)
	Deficiency	34.5 (10)	52.9 (9)	41.3 (19)	64.7 (33)	50.0 (21)	58.1 (54)	53.8 (43)	50.8 (30)	52.5 (73)

Mean corpuscular volume (fl)	High	24.1 (7)	17.7 (3)	21.7 (10)	5.9 (3)	16.7 (7)	10.7 (10)	12.5 (10)	16.9 (10)	14.4 (20)
	Standard	65.6 (19)	52.9 (9)	60.9 (28)	74.5 (38)	76.2 (32)	75.3 (70)	71.3 (57)	69.5 (41)	70.5 (98)
	Deficiency	10.3 (3)	29.4 (5)	17.4 (8)	19.6 (10)	7.1 (3)	14.0 (13)	16.2 (13)	13.6 (8)	15.1 (21)

Mean corpuscular hemoglobin (pg)	High	3.5 (1)	17.6 (3)	8.7 (4)	3.9 (2)	4.8 (2)	4.3 (4)	5.0 (4)	6.8 (4)	5.7 (8)
	Standard	75.8 (22)	41.2 (7)	63.0 (29)	54.9 (28)	73.8 (31)	63.4 (59)	61.2 (49)	66.1 (39)	63.4 (88)
	Deficiency	20.7 (6)	41.2 (7)	28.3 (13)	41.2 (21)	21.4 (9)	32.3 (30)	33.8 (27)	27.1 (16)	30.9 (43)

Mean corpuscular hemoglobin concentration (g/dL)	Normal	37.9 (11)	58.8 (10)	46.7 (21)	45.1 (23)	42.9 (18)	44.1 (41)	42.5 (34)	47.5 (28)	44.6 (62)
	Deficiency	62.1 (18)	41.2 (7)	54.3 (25)	54.9 (28)	57.1 (24)	55.9 (52)	49.5 (46)	52.5 (31)	55.4 (77)

Red cell distribution width (%)	High	3.5 (1)	29.4 (5)	13.1 (6)	11.8 (6)	9.5 (4)	10.8 (10)	8.8 (7)	15.3 (9)	11.5 (16)
	Standard	24.1 (7)	41.2 (7)	30.4 (14)	35.3 (18)	19.1 (8)	27.9 (26)	31.2 (25)	25.4 (15)	28.8 (40)
	Deficiency	72.4 (21)	29.4 (5)	56.5 (26)	52.9 (27)	71.4 (30)	61.3 (57)	60.0 (48)	59.3 (35)	59.7 (83)

Normal = number above the lower cut-off, High = number above higher cut-off value, Standard = number within the range, and Deficiency = number below the lower cut-off level. Figures in parentheses are sample number.

**Table 6:** Distribution of hematological (white blood cell) indicators by age group and sex among children in Odisha, India.

Parameter	Category	Preschool children			School age children			Pooled children		
		Boys	Girls	Total	Boys	Girls	Total	Boys	Girls	Total
WBC (thousand/mm^3^)	High	13.8 (4)	11.8 (2)	13.1 (6)	5.9 (3)	19.0 (8)	11.8 (11)	8.7 (7)	16.9 (10)	12.2 (17)
	Standard	62.1 (18)	47.1 (8)	56.5 (26)	60.8 (31)	38.1 (16)	50.6 (47)	61.3 (49)	40.7 (24)	52.5 (73)
	Deficiency	24.1 (7)	41.1 (7)	30.4 (14)	33.3 (17)	42.9 (18)	37.6 (35)	30.0 (24)	42.4 (25)	35.3 (49)

Granulocytes (%)	High	3.4 (1)	0.0 (0)	2.2 (1)	2.1 (1)	0.0 (0)	1.2 (1)	2.6 (2)	0.0 (0)	1.5 (2)
	Standard	34.5 (10)	35.3 (6)	34.8 (16)	39.6 (19)	24.3 (9)	32.9 (28)	37.7 (29)	27.8 (15)	33.6 (44)
	Deficiency	62.1 (18)	64.7 (11)	63.0 (29)	58.3 (28)	75.7 (28)	65.9 (56)	59.7 (46)	72.2 (39)	64.9 (85)

Monocytes (%)	High	13.8 (4)	0.0 (0)	8.7 (4)	6.3 (3)	5.4 (2)	5.9 (5)	9.1 (7)	3.7 (2)	6.9 (9)
	Standard	58.6 (17)	64.7 (11)	60.9 (28)	56.3 (27)	64.9 (24)	60.0 (51)	57.1 (44)	64.8 (35)	60.3 (79)
	Deficiency	27.6 (8)	35.3 (6)	30.4 (14)	37.4 (18)	29.7 (11)	34.1 (29)	33.8 (26)	31.5 (17)	32.8 (43)

Lymphocytes (%)	High	69.0 (20)	70.6 (12)	69.6 (32)	77.1 (37)	78.4 (29)	77.6 (66)	74.0 (57)	75.9 (41)	74.8 (98)
	Standard	31.0 (9)	29.4 (5)	30.4 (14)	20.8 (10)	21.6 (8)	21.2 (18)	24.7 (19)	24.1 (13)	24.4 (32)
	Deficiency	0.0 (0)	0.0 (0)	0.0 (0)	2.1 (1)	0.0 (0)	1.2 (1)	1.3 (1)	0.0 (0)	0.8 (1)

Thrombocytes (thousand/mm^3^)	High	10.3 (3)	5.9 (1)	8.7 (4)	10.4 (5)	2.7 (1)	7.1 (6)	10.5 (8)	3.7 (2)	7.6 (10)
	Standard	31.0 (9)	35.3 (6)	32.6 (15)	22.9 (11)	32.4 (12)	27.0 (23)	25.9 (20)	33.3 (18)	29.0 (38)
	Deficiency	58.7 (17)	58.8 (10)	58.7 (27)	66.7 (32)	64.9 (24)	65.9 (56)	64.5 (49)	63.0 (34)	63.4 (83)

Normal = number above the lower cut-off, High = number above higher cut-off value, Standard = number within the range, and Deficiency = number below the lower cut-off level. Figures in parentheses are sample number.

**Table 7:** Pearson's correlation coefficients (*r*) of different parameters in children of Odisha, India.

	BAZ	Hb	WBC	RBC	HCT	MCV	MCH	MCHC	THR	RDW	GRA	MON
BAZ	1.00											
Hb	0.381^‡^	1.000										
WBC	0.454	−0.232^†^	1.000									
RBC	0.288^‡^	0.871^‡^	−0.158	1.000								
HCT	0.268^†^	0.943^‡^	−0.199^*∗*^	0.940^‡^	1.000							
MCV	−0.041	0.337^‡^	−0.079	−0.019	0.281^‡^	1.000						
MCH	−0.032	0.209^*∗*^	−0.093	−0.189^*∗*^	0.066	0.757^‡^	1.000					
MCHC	−0.003	−0.006	0.133	−0.043	−0.024	0.049	0.089	1.000				
THR	0.057	−0.052	0.373^‡^	−0.033	−0.064	−0.073	−0.095	0.028	1.000			
RDW	−0.177^*∗*^	−0.367^‡^	0.112	−0.289^‡^	−0.387^‡^	−0.408^‡^	−0.192^*∗*^	−0.002	−0.046	1.000		
GRA	−0.041	−0.211^*∗*^	0.358^^‡^^	−0.090	−0.117	−0.176^*∗*^	−0.092	0.191^*∗*^	−0.048	0.101	1.000	
MON	0.135	0.277^†^	−0.001	0.216^*∗*^	0.294^‡^	0.299^‡^	0.162	−0.055	−0.069	−0.176^*∗*^	−0.176^*∗*^	1.000
LYM	0.008	0.141	−0.367^‡^	0.035	0.044	0.101	0.058	−0.181^*∗*^	0.057	−0.057	−0.961^‡^	−0.065

Significance: ^*∗*^ *p* < 0.05, ^†^ *p* < 0.01, and ^‡^ *p* < 0.001.

BAZ = BMI-for-age *z*-score, Hb = hemoglobin, RBC = red blood cell, HCT = hematocrit, MCV = mean corpuscular volume, MCH = mean corpuscular hemoglobin, MCHC = mean corpuscular hemoglobin concentration, RDW = red cell distribution width, WBC = white blood cell, GRA = granulocyte, MON = monocyte, LYM = lymphocyte, THR = thrombocyte.
